# Civic engagement and mental health trajectories in Norwegian youth

**DOI:** 10.3389/fpubh.2023.1214141

**Published:** 2023-10-19

**Authors:** Nora Wiium, Sara Madeleine Kristensen, Elisabeth Årdal, Tormod Bøe, Margarida Gaspar de Matos, Kateryna Karhina, Torill Marie Bogsnes Larsen, Helga Bjørnøy Urke, Bente Wold

**Affiliations:** ^1^Department of Psychosocial Science, University of Bergen, Bergen, Norway; ^2^Department of Health Promotion and Development, University of Bergen, Bergen, Norway; ^3^Instituto de Saúde Ambiental, University of Lisbon, Lisbon, Portugal

**Keywords:** civic engagement, youth mental health trajectories, SES, gender, Norway

## Abstract

**Introduction:**

Applying variable-centered analytical approaches, several studies have found an association between civic engagement and youth mental health. In the present study, we used a person-centered approach to explore whether civic engagement was related to optimal trajectories of mental health compared to other trajectories. We also examined how sociodemographic factors, such as socioeconomic status (SES), gender and age were related to youth mental health trajectories.

**Methods:**

Our sample comprised 675 students (aged 16–22) who had participated in three waves of data collection (*M*_age_ = 18.85, *SD* = 0.55; 43% males) in the COMPLETE project, a cluster-randomized controlled trial that involved Norwegian upper secondary schools.

**Results:**

The results revealed three trajectories of mental health (reflecting a combination of mental distress and mental well-being): optimal, intermediate, and sub-optimal. Contrary to our expectations, higher levels of civic engagement were not related to the optimal trajectory of mental health vs. other trajectories. However, we found that students who reported higher levels of SES and males were more likely to follow the optimal trajectory compared to other trajectories.

**Discussion:**

While the findings on civic engagement could be due to our measurement’s inability to capture the concept of “dugnad,” a well-established civic activity in the Norwegian society, the findings regarding the influence of SES and gender suggest that there is still more work to be done concerning the assessment and advancement of factors that can address mental health inequalities across SES and gender.

## Introduction

Youth mental health is not only a topic of scientific research interest, but it has also gained considerable public health attention. This is because of the rising rates of poor mental health among the youth and the associated devastating outcomes, such as educational difficulties, and social exclusion (1, 2). Current global statistics suggest that about 10–20% of adolescents experience poor mental health, which represents the chief cause of disability in young people and accounts for a large proportion of the global disease burden among this group (2). Moreover, among 15-19-year-old, poor mental health indicators, such as depression and anxiety are among the leading causes of illness and disability (2).

Either as a research interest or a public health concern, the focus on mental health and well-being among youth has usually adopted a negative or deficit perspective, where good mental health is repeatedly assessed as lower scores on poor mental health indicators. Consequently, prevention methods and interventions that will reduce negative developmental outcomes have largely been applied. However, with the WHO’s definition, mental health, takes on a strength-based or positive connotation. Accordingly, mental health is “a state of well-being in which every individual realizes his or her own potential, can cope with the normal stresses of life, can work productively and fruitfully, and is able to make a contribution to her or his community” (3). Based on this definition, mental health is not only a strength to have, but it is also intended to empower the individual to engage in self- and societal development. Furthermore, the important role that mental health can play in achieving global development goals has been acknowledged by its inclusion in the UN Sustainable Development Goals (SDGs). Specifically, goal 3 of the 17 SDGs reiterates world leaders’ commitment to “En47sure healthy lives and promote well-being for all at all ages” (4, p.6). With the prospect that self-, societal, and global development goals will not be achieved by youth who report excessive poor mental health, it is of utmost importance to identify factors that may hinder poor mental health as well as those that can enhance mental well-being. This identification will be necessary to inform effective mental health programs and policies for the youth.

Research indicates that risk factors, such as loneliness and isolation, bullying and substance use tend to contribute to poor mental health (5), while protective factors, such as social support and engagement in physical activity enhance mental well-being (6). Findings from earlier studies also indicate that youth civic engagement or their contribution to the community do not only protect against poor mental health (7), but it also can improve their mental well-being (8). In the present longitudinal study, we investigate the extent to which three dimensions of civic engagement: social engagement, social conscience, and civic action are associated with mental health among youth living in Norway.

Conceptually, social engagement and social conscience represent ideological forms of civic engagement, while civic action exemplifies an action-oriented form of civic engagement. Social engagement refers to young people’s interest in the activities and development of their community and the civic discussions they have with people in their immediate contexts (9). Young people engage in social conscience when they have ideologies that state that helping others or contributing to make the world a better place is a positive thing to do (10, 11). Theoretically, social conscience connotes Character, one of the thriving indicators or positive developmental outcomes proposed by Lerner et al. (11). However, in the present study, we treat it as an ideological form of civic engagement, in line with Zaff et al. (12), who adopt similar formulation in their active and engaged citizenship scale. Finally, civic action refers to the situation where young people do not only have positive ideologies but also actively engage in community development (13). Applying the Positive Youth Development perspective as a theoretical framework, with emphasis on the role contribution might have on mental health, we explore the influence of the three dimensions of civic engagement on trajectories of mental health, as indicated by mental distress and mental well-being, with gender, age, and socio-economic status as covariates.

### Positive youth development

To substitute or rather supplement the deficit approach that had characterized youth studies, Positive Youth Development emerged as a strength-based perspective at the turn of the 21st century. The PYD perspective sought more to study youth strengths (e.g., positive values and social competence) and how alignment with the resources and opportunities in youth contexts (e.g., home and school) can facilitate positive developmental outcomes and eventually, youth contribution (13). Youth who report more positive developmental outcomes are said to be thriving, and while thriving youth tend to contribute to their community, they are also less likely to report adjustment problems, such as depression, delinquency, and substance use (14, 15). Accordingly, the support and empowerment opportunities that young people experience in their interaction with caring adults and peers in youth contexts along with engagement in meaningful activities that provide possibilities for skills development, have the potential to enhance the general well-being of youth.

Consistent with Burkhard et al. (16), PYD can be conceived as a developmental process, a philosophy (where PYD initiatives incorporates ideology that makes and takes an individual through a successful transition from healthy adolescence into competent adulthood) and as instances of youth programs and organizations. Adapting the developmental process conceptualization, we investigate the theoretical part of PYD that considers the associations of contribution with adjustment problems (mental distress, in the case of the present study), in addition to well-being, a positive indicator of mental health. Accordingly, we build on earlier studies by examining associations between youth civic engagement (i.e., ideological and action forms of contribution) and different trajectories of mental health.

### Earlier research on civic engagement, sociodemographic factors, and mental health

#### Civic engagement and mental health

Earlier research indicates that civic engagement relates negatively to poor mental health, and positively to mental well-being, although the evidence has sometimes varied across groups (e.g., gender). Regarding associations with poor mental health, Landstedt et al. (17) investigated the longitudinal associations between gender, civic engagement (measured as participation in organizational activities), and depressive symptoms in a Swedish youth cohort from adolescence to adulthood (16–42 years) at four time points. Their findings indicated negative cross-sectional associations between civic engagement and depression at ages16 and 42 for females, while for males, civic engagement in adolescence (at age 16) predicted lower levels of depression symptoms in early adulthood (at age 21). Based on their findings, the authors argued that interventions that promote civic engagement in young males may be one way of addressing mental health issues at a later stage in life for this gender group.

In a study of a nationally representative sample of American students in grades 7 through 12, using data from the National Longitudinal Study of Adolescent to Adult Health, Ballard et al. (18) examined among others, the role of civic engagement in adolescence and early adulthood on health outcomes in adulthood, including depressive symptoms. Their general findings indicated robust relations of civic engagement, either as separate variables (in particular, voting and volunteering) or as a composite variable, with depressive symptoms.

Moreover, in a related longitudinal study, using the same American sample as Ballard et al.’ (18), but slightly different measures of civic engagement (e.g., community engagement, and political behaviors), Wray-Lake et al. (19) found that community engagement in adolescence and early adulthood among others, was consistently related to lower depressive symptoms over a 6-year period. Thus, Wray-Lake et al. (19) concluded that community engagement may have some positive influence on youth mental health, and that depression may deter their later civic engagement.

As for civic engagement’s positive influence on mental well-being, Wray-Lake et al. (7) investigated whether civic engagement was associated with subjective well-being as measured by positive affect and life satisfaction in a 7-day daily diary study among American college students. Civic engagement was assessed by five items: helping a person in need, protecting the environment, acting environmentally responsibly, volunteering for an organization or a cause, and making a charitable donation. Their findings showed that civic engagement was associated with higher levels of subjective well-being, on average across days, and that the association was partly mediated through the satisfaction of psychological needs. In particular, Wray-Lake et al. (7) found that civic engagement indicators, such as helping and pro-environmental behaviors, relative to volunteering and charitable giving were more strongly associated with the subjective well-being of college students.

Furthermore, in a two-study article by Chan and Mak (20), the authors first examined the empowering processes through which sociopolitical control (the belief about one’s ability in social and political systems) motivates civic engagement (e.g., helping make your city or town a better place for people to live). In this first study, they also investigated the influence of civic engagement on the emotional, psychological, and social dimension of well-being. Their sample comprised emerging adults in Hong Kong and mainland China. As results, the authors observed a positive association between sociopolitical control and civic engagement, which was sequentially associated with better psychological and social well-being, but not emotional well-being. In the second study that involved only emerging adults in Hong Kong, the authors examined the associations in a three-wave longitudinal study and confirmed the directionality of the associations found between sociopolitical control, civic engagement, and well-being. Consequently, Chan and Mak (20) reasoned that empowering emerging adults with sociopolitical control can promote civic engagement and ultimately, well-being.

#### Sociodemographic factors and mental health

Socioeconomic status and gender are some sociodemographic factors found to influence mental health. For example, in a German cohort study, children and adolescents from low SES families reported more mental health problems compared to their high SES counterparts (21). In another study involving data collected from five quadrennial survey cycles of the Health Behavior in School-aged Children study in Canada, Hammami et al. (22) examined how socioeconomic groups (measurement based on material deprivation) were associated with six health domains: daily physical activity, excess body weight, frequent physical symptoms, frequent psychological symptoms, low life satisfaction, and fair or poor self-rated health. Their findings indicated that over the 16-year period that was studied, socioeconomic differences increased in excess body weight, physical symptoms, low life satisfaction, and fair or poor health.

As for gender and its association with mental health indicators, the study by Hammami et al. (22) also indicated that girls were more likely to show poorer health than boys in all the domains that were examined except for excess body weight. Moreover, the authors found that gender differences increased over time in health domains, such as physical symptoms, psychological symptoms, and low life satisfaction. Furthermore, Campbell et al. (23) in a cross-national study of adolescents across 73 countries found that girls reported worse average mental health than boys across four measures of mental health (i.e., psychological distress, life satisfaction, eudaemonia, and hedonia) and that the gender gap was most pronounced for psychological distress and life satisfaction. Their findings also suggested that more gender equal countries tended to have larger gender gaps in mental health.

### The study context

Compared to other European countries, Scandinavian countries, especially Norway and Sweden score high on volunteering and other civic activities, with about 50% of the population of these countries engaging actively in volunteering work (24). The high levels of civic engagement found in the general population have also been observed among the youth population, especially the ideological aspect of civic engagement. Indeed, results of the 2016 International Civic and Citizenship study indicated that 14–15-year-old students in Denmark, Finland, Norway, and Sweden scored significantly higher on civic knowledge, understanding of democracy and citizenship than the international average (25). This is not surprising as education on democracy forms part of the school curriculum. However, in terms of voluntary services, not so many were actively involved, although Norwegian students more often indicated that they were members of civic organizations and participated in school democracy (26).

Focusing on Norway as the context of the present study, with the youth’s high scores in civic engagement, and the review of earlier studies that generally suggests a positive or protective effect of civic activities on mental health, it is natural to inquire how Norwegian youth are doing with respect to their mental health. In a recent study by Hafstad et al. (27), clinical levels of anxiety and depression among adolescents increased slightly from 5.5% (measured in February 2019) to 6.3% (measured during the pandemic–June 2020). As for more serious forms of mental disorders like suicide, the statistics show that suicide among children and young adolescents is rare, although among 15–24-year-olds, about 15 suicides per 100,000 inhabitants per year among males and six among females were registered between 2014 and 2018 (28). At the same time, over 90% of children and adolescents indicated in a 2019 report on quality of life and mental health published by the Norwegian Institute of Public Health (29) that they were satisfied or very satisfied with their life. With these statistics, it will be interesting to investigate whether civic engagement (measured as social engagement, social conscience, and civic action) and mental health in Norwegian youth are linked in any way as has been observed in earlier studies.

### The present study

In the present study, we examine how civic engagement in the form of ideology (social engagement, social conscience) and civic action are related to trajectories of mental health among young Norwegians. Most of the research that has been done on the topic has used a variable-centered approach, where averaged parameters have been estimated for all individuals in the study sample (30). In our study, we use a person-centered approach, which classifies individuals into distinct groups and compares them on various covariates. Identifying the characteristics of these distinct groups allows for intervention to be tailored to meet specific needs.

With the person-centered approach, individuals in one group are more like each other than individuals between groups. Accordingly, we examine how various aspects of civic engagement measured at different time points are related to trajectories of mental health distress and mental well-being. By so doing we do not only assess how mental health among young Norwegians develop over time, but we also investigate how the different trajectories differ from each other in terms of civic engagement. We hypothesize that higher levels of civic engagement would be associated with the most optimal trajectory of mental health.

Furthermore, we investigate the influence of socio-economic status and gender on mental health trajectories as well as control for age differences, which have sometimes been found to be related to both civic engagement and mental health (17, 18, 23, 31). In our study, we expect that high levels of SES and being male will be positively associated with the optimal trajectory of mental health.

## Methods

### Participants and procedure

The present study used data from the COMPLETE project (32), a cluster-randomized controlled trial that involved Norwegian upper secondary school students, from August 2016 to June 2019. The aim of COMPLETE was to improve the psychosocial learning environment and increase the completion rate in upper secondary school. All upper secondary schools in four Norwegian counties were invited to participate. A total of 17 schools participated in the study and were randomly assigned to two intervention groups (six schools in each) and one control group (five schools). All students enrolled in the first year of upper secondary school in August 2016 in the participating schools were invited to take part in the project. One intervention school dropped out after the first year. Data were collected via electronic questionnaires across four time points. However, for the present study, we used data collected across three time points in the spring semesters, in March 2017, 2018, and 2019, where data were available for all mental health measures. The initial sample size was 2,327 first year students, while the final sample included in the present study comprised 675 upper secondary school students (aged 16–22) who had participated in all three waves of data collection (*M*_*ag*e_ = 18.85, *SD* = 0.55; 43% males) (Table 1). Before the data collection, informed consent was sought from students who were assured confidentiality and anonymity of their responses. The COMPLETE study was approved by the Norwegian Center for Research Data (now Sikt—Norwegian Agency for Shared Services in Education and Research). For further details on the ethical procedure, see Larsen et al. (32).

**Table 1:** Number of participants by mean age and year of data collection.

Year	T1	T2	T3	T1, T2, and T3
Mean age (SD)	17.16 (1.16)	18.14 (1.16)	19.14 (1.16)	18.85 (0.55)
*n*	2,327	1,729	1,127	675

### Measures

#### Mental distress

Mental distress was measured with the Norwegian short version of the Symptom Check List-90-R, which contains five items that reflect anxiety and depression (33). Participants responded to how bothered or distressed they had been in the last 14 days on a scale ranging from 1 = *not at all* to 4 = *very much*. Sample items are “Feeling tense or uneasy” and “Feeling blue and sad.” Previous research indicates good internal consistency with high Cronbach alpha values (> 0.83) (33–36). For the present study, the Cronbach’s alphas for the three waves of data were 0.88, 0.88, and 0.90, respectively, indicating high reliability of the measure on mental distress.

#### Mental well-being

To measure students’ mental well-being, we used the Norwegian short version of the Warwick-Edinburgh Mental Well-being Scale (SWEMWBS) (37–39), that consists of seven items. Sample items include, “I’ve been feeling optimistic about the future” and “I’ve been feeling useful.” Participants responded to how they had “felt” or “thought” during the last 14 days on a Likert-scale from 1 = *none of the time* to 5 = *all of the time*. The scale has shown good internal consistency in adolescent samples with Cronbach alphas ranging from α ≥ 0.78 to 0.88 (38, 40, 41). The Cronbach’ alphas across the three waves of data collection for the current study were all 0.91, indicating high reliability of the mental well-being scale.

#### Civic engagement

Civic engagement was measured with three scales capturing *social engagement, social conscience,* and *civic action*. *Social engagement* was measured with seven items at T1 and T3, adopted from the IEA International Civic and Citizenship Education Study 2016 (42). Students were asked to indicate how often they were involved in a series of activities on a scale from 1 to 4 [1 = Never or hardly ever, 2 = Monthly (at least once a month), 3 = Weekly (at least once a week), and 4 = Daily or almost daily]. A sample item is “Reading the newspaper to inform yourself about national and international news.” The Cronbach’s alpha values for the two time points were 0.84 and 0.85, respectively.

*Social conscience* was measured at all three time points: T1, T2, and T3, with the short form of the *Character* scale of the 5Cs model of Positive Youth Development (10). Although reflecting character, similar items have also been used to assess an ideological dimension of civic engagement (12). The scale consists of six items, where participants were asked to indicate how important a series of actions, for example, was to them as a person, on a scale from 1 = not important to 5 = very important. Sample items include “Helping to make the world a better place to live in.” The scale has shown good internal consistency in adolescent samples (α 0.89–0.92) (10, 13). For the current study, Cronbach’s alphas were 0.91, 0.91, and 0.93, respectively.

*Civic action* was measured at T2 and T3 with the *Action* subscale of the *Contribution* scale by Geldhof et al. (10), consisting of six items. Students were asked to specify how often they engaged in a series of actions and respond on either a scale from 1 to 5 or 1 to 6, with slightly different response categories depending on the action. Sample items include “Help a neighbor” (response scale: 1 = *Never*, 2 = *Rarely*, 3 = *Occasionally*, 4 = *Often*, and 5 = *Very often*) and “Contribute as a volunteer (for example in a sports club, youth club, band or NGOs like the Red Cross etc.)” (Response scale: 1 = *Never*, 2 = *Once a month or less*, 3 = *A couple of times a month*, 4 = *Once a week*, 5 = *A few times a week*, and 6 = *Every day*). While no previous study could be identified assessing the internal consistency of the action subscale, previous research has found the overall Contribution scale to have good internal consistency (10). In the present study, the three items with 1–6 response categories were recoded to 1–5, where 2 = *Once a month or less* and 3 = *A couple of times a month* were treated as one response category. Cronbach’s alphas for the current study were 0.68 and 0.58, respectively.

#### Sociodemographic factors

Three sociodemographic factors were examined in the study: socioeconomic status (SES), gender, and age. To measure SES, we used a single-item measure of perceived family wealth (43), asking “How well off is your family?” The participants responded on a five-point Likert-scale ranging from 1 = *not at all well off* to 5 = *very well off.* This subjective measure of SES has been widely used in large scale surveys involving adolescents [e.g., (44)].The influence of *gender* (male coded as 0 and female coded as 1) was also assessed while *age* (number of years) was treated as a control variable. In addition, we checked the effects of intervention condition (one control group and two intervention groups) in our preliminary analysis. We used the control group as a reference group and created two dummy variables (one for each intervention group).

### Data analysis

Descriptive analysis was conducted on each of the study variables (i.e., three sociodemographic variables, three dimensions of civic engagement, and the two mental health outcomes) to ascertain their pattern of distribution as well as the bivariate associations between the variables. Intervention condition was excluded from the analysis after the preliminary analysis did not reveal any statistically significant associations with the study variables. Longitudinal measurement invariance (i.e., configural invariance, metric invariance, and scalar invariance) was then estimated to ensure that meaningful comparisons can be made across the three time points (i.e., T1, T2, and T3). Configural invariance is when the items load onto the latent factor (each of the civic engagement and mental health dimensions) in the same manner across time. For metric invariance, the factor loadings of the items measuring civic engagement and mental health are the same across time points. For scalar invariance, the factor loadings, as well as the intercepts, are the same across time.

Following the longitudinal measurement invariance, latent class growth analysis (LCGA) was used to estimate trajectories of mental health (mental distress and mental well-being) where different trajectories were identified from the data. In LCGA, the variance and covariance estimates of the growth factors are fixed to 0, a statistical procedure that does not allow for variation within classes (45). Relevant sociodemographic variables (gender, age, and SES) and the three dimensions of civic engagement were included in model specification as covariates or as independent variables of class membership. The most optimal number of classes was selected based on a theoretical understanding of the trajectories as well as by using statistical criteria, such as the Akaike information criterion (AIC), the Bayesian Information Criterion (BIC) value (46), Lo et al. (47) Lo–Mendell–Rubin adjusted likelihood ratio test (LMR-LRT) statistic, and the bootstrap likelihood ratio test (BLRT) statistic. Following the proposal of Nylund et al. (46), a non-significant *p* value from the LMR test was used as a stopping rule for the number of classes that should be derived. However, as the LMR test might overestimate the number of classes, the test was followed by an inspection of other fit indices, such as the BLRT and BIC, which have been found to perform best (46). In general, smaller BIC as well as AIC values are preferable, although the latter criterion has not always been found reliable. Entropy ranging between 0 and 1, where 1 is the best classification, was used to assess classification accuracy of the individuals.

The LCGA analyses were examined for non-convergence and local maxima and treated accordingly by increasing the number of random starting values. Except for the descriptive analysis, which was conducted using the Statistical Product and Service Solutions (SPSS statistical program), all other analyses were run using the Mplus statistical program, version 8 (48). The different models were estimated using the full information maximum likelihood (FIML) estimator with robust standard errors (MLR) where the civic engagement and mental health dimensions were treated as mean scores. As an estimation method that is used to handle missing cases, FIML uses all available data and operates by estimating a likelihood function for each case based on the variables present in the dataset.

## Results

### Dropout analysis

Dropout analysis was conducted by comparing scores of mental distress and mental well-being to determine whether those who were included in the statistical analysis (i.e., those who participated in all three data collections) differed from those who were excluded (i.e., participants who did not participate in all three data collection). About 22% of the participants (*n* = 675) were involved in all three data collections. Participants who were included in the study and those who were not, did not differ on their mental health scores except for mental well-being at T1, where the former attained a higher mean score (*M* = 3.60, *SD* = 0.78) compared to the latter (*M* = 3.44, *SD* = 0.89), *t*(2,180) = −3.95, *p* < 0.001; and mental distress at T2, where the former reported again higher mean scores (*M* = 1.95, *SD* = 0.79) relative to the latter (*M* = 1.83, *SD* = 0.82), *t*(1,667) = −3.00, *p* < 0.01. In addition, the proportion of females in the group of participants with all three datasets was greater than that of the group that did not have all the data (57 vs. 40%, chi-square = 58.479, *p* < 0.001).

### Descriptive analysis of study variables

For the study sample, mean scores (maximum of 4) for social engagement measured at T1 and T3 were 2.52 and 2.68, respectively, suggesting that responses on this ideological dimension of civic engagement were mostly between “once a month” and “every week” (Table 2). Mean scores (maximum of 5) for social conscience (another ideological dimension), which was measured at T1, T2, and T3, were 4.03, 4.03, and 4.11, respectively. Participants’ responses on this second dimension of civic engagement were thus between “quite important” and “very important.” Finally, for civic action (the more action-oriented dimension of civic engagement), the mean scores (2.09 and 2.21, out of a maximum of 5) indicated that participants’ responses to the measure at T2 and T3 were between “rarely” and “occasionally” or between a few times in a month and once in a week, depending on the response categories that were used. As for the mental health dimensions, the mean scores at T1, T2, and T3 were 1.87, 1.95, and 2.07 (maximum of 4) for mental distress and 3.60, 3.53, and 3.50 (maximum of 5) for mental well-being, respectively. Thus, for mental distress, the responses centered around “a bit bothered” while for mental well-being the responses were between “sometimes” and “often.”

**Table 2:** Descriptive and correlation analysis of civic engagement, sociodemographic factors, and mental health.

Study variables	1	2	3	4	5	6	7	8	9	10	11	12	13	14	15	16
1. Gender	-	−0.05	−0.12**	−0.05	0.01	0.34**	0.28**	0.31**	0.03	−0.02	0.37**	0.32**	0.31**	−0.13**	−0.16**	−0.23**
2. Age		-	−0.03	0.04	−0.05	−0.01	−0.02	−0.04	−0.01	0.00	0.00	0.05	−01	0.06	−0.02	−0.04
3. SES			-	0.05	0.06	0.06	0.03	0.01	−0.08	0.01	−0.20**	−0.12**	−0.15**	0.20**	0.13**	0.17**
4. T1_ Social engagement				-	0.59**	0.14**	0.14**	0.10*	0.19**	0.19**	0.01	0.05	−0.03	0.18**	0.15**	0.18**
5. T3_ Social engagement					-	0.15**	0.15**	0.16**	0.21**	0.23**	−0.03	−0.04	−0.09*	0.16**	0.23**	0.24**
6. T1_Social conscience						-	0.67**	0.55**	0.22**	0.18**	0.16**	0.13**	0.12**	0.07	0.03	−0.03
7. T2_Social conscience							-	0.67**	0.23**	0.23**	0.14**	0.09*	0.07	0.07	0.09*	0.04
8. T3_Social conscience								-	0.20**	0.24**	0.12**	0.09*	0.05	0.08*	0.08*	0.12**
9. T2_Civic action									-	0.59**	0.00	−0.02	−0.06	0.09*	0.09*	0.12**
10. T3_Civic action										-	−0.04	0.04	−0.07	0.14**	0.15**	0.21**
11. T1_Mental distress											-	0.64**	0.56**	−0.53**	−0.45**	−0.40**
12. T2_Mental distress												-	0.66**	−0.38**	−0.59**	−0.48**
13. T3_ Mental distress													-	−0.38**	−0.49**	−0.65**
14. T1_Mental well-being														-	0.56**	0.46**
15. T2_Mental well-being															-	0.61**
16. T3_Mental well-being																-
Descriptive analysis																
Range	0–1	16–22	1–5	1–4	1–4	1–5	1–5	1–5	1–5	1–5	1–4	1–4	1–4	1–5	1–5	1–5
Mean	0.57	18.85	3.82	2.52	2.68	4.03	4.03	4.11	2.09	2.21	1.87	1.95	2.07	3.60	3.53	3.50
S.D.	0.50	0.55	0.78	0.61	0.66	0.81	0.80	0.81	0.54	0.56	0.77	0.79	0.81	0.78	0.81	0.79
Cronbach’s alpha α	-	-	-	0.84	0.85	0.91	0.91	0.93	0.68	0.58	0.88	0.88	0.90	0.91	0.91	0.91

^*^p < 0.05; ^**^p < 0.05.

In the correlation analysis (Table 2), weak correlations were observed between the civic engagement dimensions and mental health with the weakest between civic action at T2 and mental distress at T1 (*r* = 0.00) and the strongest between social engagement at T2 and mental well-being at T3 (*r* = 0.24, *p* < 0.01). Correlations between the sociodemographic variables, civic engagement and mental health were weak to moderate. More specifically, female participants relative to males, were more likely to report poor mental health and less mental well-being. There were no significant correlations between age and the mental health dimensions. SES was negatively associated with mental distress and positively associated with mental well-being (Table 2).

### Measurement invariance

To ensure meaningful comparison across time, longitudinal measurement invariance was estimated for civic engagement and mental health. For the civic engagement variables, scalar invariance was achieved for social conscience, while partial scalar invariance was attained for social engagement and civic action when the intercepts of two items measuring the former and four items measuring the latter were allowed to vary across time. Thus, it is possible to make a mean comparison for these two civic engagement measures, albeit it should be done with caution. As for the two dimensions of mental health, scalar invariance was achieved for both measures, allowing for meaningful comparisons across time (Table 3).

**Table 3:** Longitudinal invariance models for civic engagement and mental health indicators.

	Model fit indices			
	*χ^2^ (df)*	*RMSEA*	*90% CI RMSEA*	*CFI/TLI*
Social engagement				
Configural invariance	287.435 (65)	0.071	0.06 3–0.080	0.944/0.921
Metric invariance	306.961 (72)	0.070	0.062–0.078	0.940/0.925
Scalar invariance	413.052 (79)	0.079	0.072–0.087	0.915/0.905
*Partial scalar invariance*	*367.179 (77)*	*0.075*	*0.067–0.082*	*0.926/0.913*
Social conscience				
Configural invariance	470.821 (114)	0.068	0.062–0.075	0.948/0.930
Metric invariance	499.128 (126)	0.066	0.060–0.072	0.946/0.934
*Scalar invariance*	*541.529 (138)*	*0.066*	*0.060–0.072*	*0.941/0.935*
Civic action				
Configural invariance	91.770 (43)	0.041	0.029–0.053	0.959/0.937
Metric invariance	113.438 (49)	0.044	0.034–0.055	0.946/0.927
Scalar invariance	244.742 (55)	0.071	0.062–0.081	0.841/0.809
*Partial scalar invariance*	*118.931 (51)*	*0.044*	*0.034–0.055*	*0.943/0.926*
Mental distress				
Configural invariance	151.508 (72)	0.040	0.031–0.049	0.984/0.977
Metric invariance	172.503 (82)	0.040	0.032–0.049	0.982/0.977
*Scalar invariance*	*237.257 (92)*	*0.048*	*0.041–0.056*	*0.971/0.967*
Mental well-being				
Configural invariance	567.140 (165)	0.060	0.055–0.066	0.944/0.929
Metric invariance	583.077 (179)	0.058	0.053–0.063	0.944/0.934
*Scalar invariance*	*619.934 (193)*	*0.057*	*0.052–0.062*	*0.941/0.935*

### Determining the optimal number of latent class of mental health

In Table 4, the findings from the LCGAs that were undertaken with one to four-class solutions are presented. Based on a theoretical understanding of the trajectories and statistical criteria, we stopped increasing the number of classes at four as the fit of the four-class model was not significantly better than the fit of the three-class model. This decision was in accordance with the non-significant LMR test (*p* = 0.0519). Moreover, the four-class model did not add any distinctive features over the three-class model as two of its classes only differed quantitatively (on the level of mental health over time) rather than qualitatively (on the shapes of the growth curves). The three-class model was chosen as the most optimal because the BIC value was the smallest for the three-class model and the BLRT test revealed a significant difference between the two and three-class models. In addition, the three-class model was theoretically more meaningful than the two-class model. The number (and proportion) of participants belonging to the first, second, and third latent classes (or trajectories) were 280 (41.5%), 282 (41.8%), and 113 (16.7%), respectively, while the respective estimated probabilities of belonging to the trajectories were 92.8, 89.0, and 93.5%, with an entropy of 0.805.

**Table 4:** Fit Indices for latent class growth analysis of mental health (mental distress and mental well-being).

Number of classes	Fit indices					
	BIC	Adjusted BIC	AIC	Entropy	LMR-LRT *p* values	BLRT
1	9422.549	9390.798	9377.402	-	-	-
2	8205.366	8157.739	8137.645	0.858	0.0000	0.0000
3	7915.435	7851.933	7825.140	0.805	0.0010	0.0000
4	7822.342	7742.964	7709.474	0.755	0.0519	0.0000

BIC, Bayesian information criterion; AIC, Akaike information criterion; LMR–LRT, Lo–Mendell–Rubin adjusted likelihood ratio test; BLRT, Bootstrap likelihood ratio test.

### Latent profiles of mental health and associations with civic engagement and sociodemographic factors

**Figure 1:**
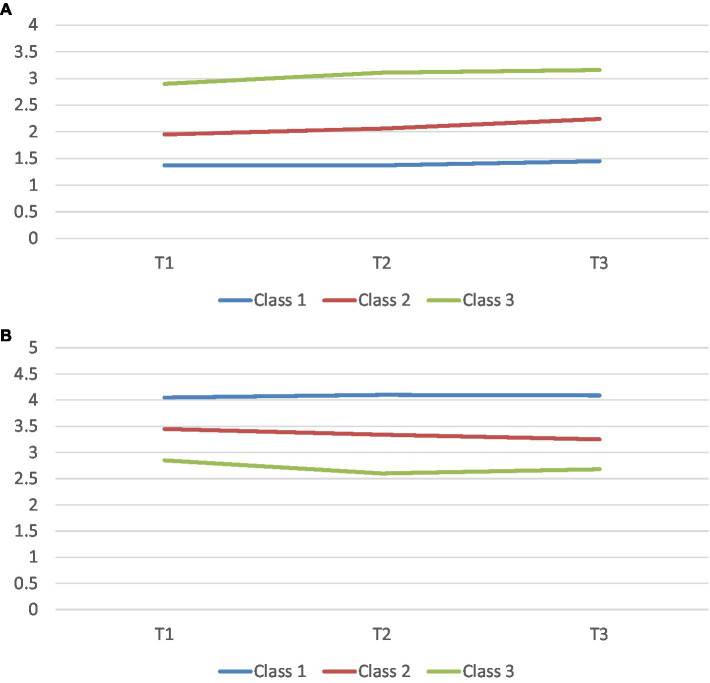
**(A,B)** Latent class growth analyses of mental distress and mental well-being, respectively: A three-wave data (mean scores); Class 1—Optimal trajectory; Class 2—Intermediate trajectory; and Class 3—Sub-optimal trajectory.

The first trajectory of mental health (represented by the blue line) was labeled as the optimal trajectory due to their lower scores on mental distress and higher scores on mental well-being (Figures 1A,B). The mean scores on mental distress at T1, T2, and T3 were 1.37, 1.37, and 1.45, while for mental well-being, the mean scores were 4.05, 4.10, and 4.09, respectively. The average initial starting point (intercept mean = 1.36) for mental distress, as well as the change over time (slope mean = 0.04), were significant at *p* = 0.05–0.001, while only the intercept mean for mental well-being was significant (4.06, *p* < 0.001). Thus, while mental distress increased marginally for the optimal trajectory, mental well-being remained relatively high and stable across time.

We labeled the second trajectory (indicated by the red line) as the intermediate trajectory because of their average scores on both mental distress and mental well-being. Mental distress for the trajectory increased slightly from T1 (mean = 1.95) to T3 (mean = 2.24), while mental well-being decreased marginally from T1 (mean = 3.45) to T3 (mean = 3.25). The intercept and slope means, which were significant at *p* = 0.01, were 1.93 and 0.15 for mental distress and 3.45 and − 0.10 for mental well-being, respectively. Accordingly, with an average initial starting point that differed significantly, an increase in mental distress and a decline in mental well-being over time were observed in the intermediate trajectory.

Finally, we labeled the third trajectory of mental health (represented by the green line) as the sub-optimal trajectory owing to the relatively higher scores on mental distress and lower scores on mental well-being. The mean scores on mental distress increased from T1 (mean = 2.90) to T3 (mean = 3.16), while the mean scores on mental well-being remained stable at average levels at 2.85, 2.60, and 2.68, respectively (Figures 1A,B). The intercept and slope means, which were significant at *p* = 0.05–0.001, were 2.93 and 0.12 for mental distress and 2.79 and − 0.09 for mental well-being. Thus, like the intermediate trajectory, an increase over time and a decline was observed for mental health and mental well-being, respectively, although the changes over time appeared to be somewhat greater for the intermediate trajectory.

In Table 5, findings from a series of multinomial logistic regressions are presented. Using the optimal trajectory of mental health as the reference group, individuals in the intermediate trajectory did not differ significantly from the optimal trajectory in their level of civic engagement, not on the ideological nor on the action-reoriented dimension. There was also no difference in civic engagement scores between the intermediate and sub-optimal trajectories. However, we found that the optimal and intermediate trajectories differed significantly on gender and SES, where members of the intermediate trajectory of mental health were more likely to be females or to report lower scores on SES (unstandardized estimate = 0.99, *p* < 0.01 and unstandardized estimate = −0.77, *p* < 0.01, respectively). The equivalent proportions of females in the optimal and intermediate trajectories were 35.2 and 69.5%. Similarly, when the optimal trajectory of mental health was compared to the sub-optimal trajectory, members of the latter were more likely to be females or participants who reported lower scores on SES (unstandardized estimate = 1.54, *p* < 0.01 and unstandardized estimate = −0.99, *p* < 0.01, respectively). The corresponding proportions of females in the optimal and sub-optimal trajectories were 35.2 and 81.4%. With the intermediate trajectory of mental health as the reference, the intermediate and sub-optimal trajectories did not differ significantly on any of the sociodemographic variables. There was also no difference in their scores regarding the civic engagement dimensions (Table 5).

**Table 5:** Associations between trajectories of mental health indicators, sociodemographic factors and civic engagement: multinomial logistic regressions (*N* = 675).

	Trajectory 1 (Optimal) as Reference			
	Trajectory 2 Intermediate		Trajectory 3 Sub-optimal	
	*Estimate*	*p value*	*Estimate*	*p value*
Gender	0.99	0.001	1.54	0.000
Age	−0.17	0.365	−0.48	0.152
SES	−0.77	0.000	−0.99	0.000
T1_ Social engagement	−0.24	0.694	−0.16	0.761
T3_ Social engagement	0.08	0.881	−0.17	0.755
T1_Social conscience	0.09	0.623	0.33	0.139
T2_Social conscience	−0.44	0.100	−0.21	0.338
T3_Social conscience	0.61	0.086	0.14	0.475
T2_Civic action	−0.14	0.669	−0.13	0.879
T3_Civic action	0.21	0.454	0.38	0.504
	Trajectory 2 (Intermediate) as Reference			
	Trajectory 1 Optimal		Trajectory 3 Sub-optimal	
	*Estimate*	*p value*	*Estimate*	*p value*
Gender	−0.99	0.001	0.56	0.087
Age	0.17	0.365	−0.31	0.353
SES	0.77	0.000	−0.22	0.237
T1_ Social engagement	0.24	0.694	0.08	0.763
T3_ Social engagement	−0.08	0.881	−0.24	0.279
T1_Social conscience	−0.09	0.623	0.24	0.328
T2_Social conscience	0.44	0.100	0.24	0.434
T3_Social conscience	−0.61	0.086	−0.47	0.225
T2_Civic action	0.14	0.669	0.01	0.989
T3_Civic action	−0.21	0.454	0.17	0.769

Gender: (0) male, (1) female; ^**a**^Trajectory 1 has the same estimates as trajectory 2 when the latter is treated as the reference group except that estimates have a positive sign instead of a negative sign and vice versa.

## Discussion

### Main findings

Positive associations of civic engagement with youth mental health have been observed in earlier studies. These studies have mainly assessed the associations using variable-centered analytical approaches. In the present study, we used a person-centered approach to investigate different trajectories of mental health and to examine how these trajectories were associated with diverse dimensions of civic engagement. We also examined the influence of SES and gender on the different trajectories. Based on theoretical and analytical appraisal, we identified three trajectories of mental health: an optimal trajectory (with lower scores on mental distress and higher scores on mental well-being), an intermediate trajectory (with average scores on both mental distress and mental well-being), and a sub-optimal trajectory of mental health (with relatively higher scores on mental distress and lower scores on mental well-being). Contrary to our expectation, higher levels of civic engagement were not associated with the optimal trajectory of mental health in comparison to the other trajectories. However, we found higher levels of SES to be associated with the optimal trajectory when compared to the two other trajectories. Moreover, for gender, we also found that females relative to males, were more likely to belong to the intermediate and sub-optimal trajectories of mental health.

### Civic engagement and trajectories of mental health

Findings from our person-centered analytical approach did not reveal any significant differences in civic engagement (ideology or action) between the three mental health trajectories. However, findings from our correlation analysis (a variable-centered analysis) indicated statistically significant concurrent and longitudinal correlations between some indicators of civic engagement and mental health (especially mental well-being). These findings, albeit reflecting weak associations, align with earlier findings (7, 17). The findings are also consistent with the PYD theoretical assumption that suggests that youth contribution (civic engagement in our case) is associated with less adjustment problems, such as mental distress (11). Moreover, in agreement with the PYD theoretical perspective, we also found positive correlations between civic engagement and mental well-being. Accordingly, from our empirical evidence, there is an indication that participating in civic engagement may enhance mental health. Lerner et al. (11) argue that youth contribution to community is usually a signal that youth are thriving. Thriving, in turn, is a function of an adaptive interaction between youth strengths and resources in their contexts (49). Youth that have experienced this adaptive interaction are therefore likely to have a buffer against mental distress as well as a resourceful environment that can enhance their mental well-being. However, contrary to our expectations, the optimal trajectory of mental health that reflects mental well-being over time did not appear to be due to youth adaptive interaction within civic engagement.

The findings that engagement in civic activities predicts better mental health has been quite consistent in the literature. However, earlier research on how civic engagement predicts trajectories of mental health over time is sparse. Indeed, to the best of our knowledge, our study is the first to investigate this prediction. While we did not expect the findings to be different when assessing mental health as trajectories, there is a possibility that civic engagement among youth in Norway (the study context) involves more than the indicators we have focused on. Scandinavian countries in general, and Norway in particular, are known for their engagement in civic activities compared to other European countries (24).

In the Norwegian society, the concept of “dugnad” as a civic activity is well established. It can be anything from getting together to help friends move house, cleaning the streets with neighbors, to organizing events in the local community. Dugnad is particularly important when a major task needs to be undertaken in a short time and there are limited funds to get the task done. By mobilizing many people, a lot of work can be done in one afternoon or over a weekend. Following a day or several days of dugnad a joint informal meal is often provided by the receiver or organizer of the dugnad (a local NGO, an individual or neighborhood initiative), underscoring the importance of the social part of the dugnad. Thus, a core element of dugnad and its value in the Norwegian society is the sense of unity and social responsibility, reflecting opportunities and qualities that can mitigate mental distress as well as improve mental well-being. These unique forms of civic engagement in Norway may not have been adequately captured by the standardized assessment tools used in the present study.

Furthermore, while in-person engagement in community development is important, globalization, technological advancement, and modernization might have changed the way young people engage in civic activities. A United Nations Children’s Fund (UNICEF) report on digital civic engagement suggests that digital venues may offer many of today’s youth opportunities to engage in civic activities at different societal levels that could not be afforded by traditional forms (50). The UNICEF report indicates that across 11 countries, between 43 and 64% of 9–17-year-old search for news online, while 12–27% discuss political problems online. Indeed, as the digital and virtual context is steadily becoming a vital arena where young people learn, play, and grow, proposals have been made to extend top theoretical models like Bronfenbrenner’s bioecological theory (51) that outlines the significance of different environments in human development, to add in and address digital space as a critical context in youth development (52). Like the youth sample described in the UNICEF report, it is possible that Norwegian youth’s engagement in civic activities is mostly in digital space, an avenue that was not clearly considered in our study.

### Sociodemographic factors and trajectories of mental health

The findings of the current study indicated that a subjective evaluation of the youth’s SES was significantly associated with the optimal trajectory of mental health, in comparison with the intermediate and sub-optimal trajectories. Consequently, youth reporting higher levels of SES were more likely to report higher levels of mental health over time. Earlier studies using variable-centered analytical procedures have indicated a negative association between low SES and poor mental health (21, 53). Moreover, using an SES measure that comprised multiple indicators (e.g., family affluence scale and parental employment), Lowthian et al. (54) found that mental well-being was highest in secondary school students from higher affluence (SES) families compared to those belonging to four other lower SES trajectories. Our findings suggest that youth who report high SES do not only report better mental health, but they also tend to do so over a sustained period of time. Having access to relations and empowering environment that can offer resources and opportunities are some of the factors that appear to differentiate high SES youth from low SES youth in terms of mental well-being (11, 54). In our findings, the significant role of SES in mental health does not appear to be linked to resources and opportunities provided within civic engagement as these two factors were not related.

Other sociodemographic factors, such as age and gender were examined in the analysis, and while age had no significant associations with mental health, significant correlations of gender were found with mental distress, and mental well-being, where females were more likely to report higher scores on mental distress, but lower scores on mental well-being. These are findings that have also been recounted in earlier studies (17, 23). Moreover, our findings indicated that males compared to females were more likely to belong to the optimal trajectory of mental health. With males and females differing to a very small extent on civic engagement (only on social conscience), the gender difference in mental health cannot be primarily attributed to their differential engagement in civic activities. In a recent cross-national study that observed a sharper decrease in life satisfaction with age (between 10 and 16) among females vs. males, determining factors, such as appearance concern, school pressures, screen time, and technology usage were proposed, with the argument that these factors tend to have larger effects on female adolescents’ mental health (55).

### Limitations

In addition to our measurement of civic engagement, which may not have sufficiently depicted Norway’s concept of dugnad as well as modern digital forms of civic engagement, other limitations related to methodology need to be addressed. Social conscience, one of our ideological dimensions of civic engagement was assessed using items that Lerner et al. (11) have used to measure “Character,” a thriving indicator within PYD. Even though similar items have been used by Zaff et al. (12) to denote civic engagement, civic engagement within PYD is a form of contribution and thus, supposed to be theoretically different from the concept of thriving. In future studies, scales that may reflect more than their intended underlying factor should be evaluated well before their use.

Moreover, the measurement of the action-oriented scale of civic engagement may have some limitations. The measures like “Help a neighbor” or “Contribute as a volunteer” only tap into services that young people render without any form of payment. It is possible that some participants volunteered in some activities and received honorary, a token for their services rather than full payment but could not register it in the questionnaire. At the same time, civic action may not take place as frequently as the ideological forms of civic engagement as reflected in the low mean scores and Cronbach’s alpha. All these are measurement issues that need to be addressed in further research to ascertain an accurate assessment as well as influence of civic action.

Furthermore, although a representative sample of upper secondary school students were involved in the study from the beginning, our inclusion of students who only had data from the three waves of data collection might have altered the sample representativeness. Besides, while there were very few differences between those who were included in the study and those who were not, the two groups differed on mental well-being at T1and mental distress at T2, where those included in the study reported higher mean scores. The proportion of females in the study sample was also greater. These group differences that were due to attrition, might probably have biased our findings. Attrition can be a problem in longitudinal studies and is not always easy to address due to the ethical necessity of voluntary participation. However, a follow-up on participants to understand their reasons for dropping out, where possible, can help prevent some of the problems related to attrition.

### Implications for research, policy, and practice

Despite the limitations of the present study, our findings, emerging from rigorous data analysis, have some implications for research, policy, and practice. For research, our findings suggest that different categories of youth tend to follow different trajectories of mental health that reflect different levels of mental distress and mental well-being. A deeper understanding of these trajectories and their determinants can be of research interest. Determinants like socioeconomic status and gender that were found to be related to the mental health trajectories, as well as appropriate measures of civic engagement that capture unique forms of engaging in civic activities, such as the Norwegian “dugnad,” can be targeted in research to understand their effects and underlying mechanisms.

As for policy, the consistency between the current findings and earlier ones regarding the poorer mental health of youth from low SES families and females, continues to trigger the need for youth policies that can align with UN SDGs’ objectives to address these inequalities. With the devasting effects of poor mental health and our findings that about 17% of upper secondary school students belong to a sub-optimal trajectory, school polices that contain both preventive and promotion strategies remain a priority in addressing the negative and positive aspects of students’ mental health.

Finally, our findings also have some implications for practice. School interventions that incorporate different preventive and promotion strategies in their health programs and activities to meet the needs of different clusters of students can be useful to address the influence of socioeconomic status and gender on mental health. While the influence of civic engagement on mental health over time was not that apparent, the indication of a concurrent association between the two factors, in line with earlier studies, may suggest that encouraging civic activities in the school can enhance students’ mental health to some extent.

## Conclusion

Earlier studies using variable-centered procedures have increasingly found positive associations between indicators of civic engagement and youth mental health. However, in the present study, although we found some significant associations between the two factors in univariate analysis, our findings from a person-centered approach did not confirm any significant differences between the optimal, intermediate, and sub-optimal trajectories of mental health in terms of civic engagement (either as an ideology or action-oriented). This is surprising, considering that Norway like other Scandinavian countries, is noted for its strong participation in civic activities, which in our expectation, should translate to better mental health over time. While this finding could be related to our measurement of civic engagement, which possibly did not adequately capture the concept of “dugnad,” a well-established civic activity in the Norwegian society, both civic engagement and mental health may be impacted by trends in globalization, modernization, and individualization, causing them to play out differently. Notwithstanding, the findings that high SES and males are more likely to belong to optimal trajectories of mental health overlap with earlier conclusions and suggest that there is still more work to be done concerning the assessment and advancement of factors determining the well-being of youth belonging to low SES and the female gender, a step that can help reach the aims of UN SDGs, in general, and goal 3, in particular.

## Data availability statement

The raw data supporting the conclusions of this article will be made available by the authors, without undue reservation.

## Ethics statement

The studies involving humans were approved by Norwegian Center for Research Data. The studies were conducted in accordance with the local legislation and institutional requirements. All participants provided their informed consent to participate in this study. Written informed consent from parents/guardians was not required as all participants were above the age of 16 at the time of participation.

## Author contributions

NW and SK were responsible for conceptualization, original draft writing, methodology, software, formal analysis, and revisions. TL, HU, and EÅ coordinated the data collection, TL, HU, EÅ, TB, MG, KK, and BW contributed to conceptualization, methodology, and revisions. All authors contributed to the article and approved the submitted version.

## References

[1] Drew N Funk M Tang S Lamichhane J Chávez E Katontoka S. Human rights violations of people with mental and psychosocial disabilities: an unresolved global crisis. Lancet. (2011) 378:1664–75. doi: 10.1016/S0140-6736(11)61458-X, PMID: 22008426

[2] World Health Organization (2021). Adolescent mental health. Available at: https://www.who.int/news-room/fact-sheets/detail/adolescent-mental-health (Accessed September 25, 2022).

[3] World Health Organization (2004). Promoting mental health: Concepts, emerging evidence, practice (summary report). World Health Organization, Geneva. Available at: https://www.who.int/mental_health/evidence/en/promoting_mhh.pdf (Accessed June 1, 2021).

[4] United Nations (2017). Work of the statistical commission pertaining to the 2030 agenda for sustainable development. [Resolution adopted by the General Assembly on 6 July 2017]. Available at: https://undocs.org/A/RES/71/313 (Accessed April 29, 2023).

[5] Ingram I Kelly PJ Deane FP Baker AL Goh M Raftery DK. Loneliness among people with substance use problems: a narrative systematic review. Drug Alcohol Rev. (2020) 39:447–83. doi: 10.1111/dar.13064, PMID: 32314504

[6] Miller KJ Mesagno C McLaren S Grace F Yates M Gomez R. Exercise, mood, self-efficacy, and social support as predictors of depressive symptoms in older adults: direct and interaction effects. Front Psychol. (2019) 10:2145. doi: 10.3389/fpsyg.2019.02145, PMID: 31632315 PMC6761306

[7] Wray-Lake L DeHaan CR Shubert J Ryan RM. Examining links from civic engagement to daily well-being from a self-determination theory perspective. J Posit Psychol. (2019) 14:166–77. doi: 10.1080/17439760.2017.1388432

[8] Fenn N Robbins ML Harlow L Pearson-Merkowitz S. Civic engagement and well-being: examining a mediational model across gender. Am J Health Promot. (2021) 35:917–28. doi: 10.1177/08901171211001242, PMID: 33739159

[9] Schulz W. Ainley J. Fraillon J. Losito B. Agrusti G. (2016). IEA international civic and citizenship education study 2016 assessment framework. Springer Nature.

[10] Geldhof GJ Bowers EP Boyd MJ Mueller MK Napolitano CM Schmid KL. Creation of short and very short measures of the five Cs of positive youth development. J Res Adolesc. (2014) 24:163–76. doi: 10.1111/jora.12039

[11] Lerner RM Wang J Hershberg RM Buckingham MH Harris EM Tirrell JM. Positive youth development among minority youth: a relational developmental systems model In: Cabrera NJ Leyendecker B, editors. Handbook on Positive Development of Minority Children and Youth. Netherlands: Springer Science + Business Media (2017). 5–17.

[12] Zaff J Boyd M Li Y Lerner JV Lerner RM. Active and engaged citizenship: multi-group and longitudinal factorial analysis of an integrated construct of civic engagement. J Youth Adolesc. (2010) 39:736–50. doi: 10.1007/s10964-010-9541-6, PMID: 20473560

[13] Lerner RM Lerner JV Almerigi JB Theokas C Phelps E Gestsdottir S. Positive youth development, participation in community youth development programs, and community contributions of fifth-grade adolescents: findings from the first wave of the 4-H study of positive youth development. The J Early Adolesc. (2005) 25:17–71.

[14] Gestsdottir S Bowers E von Eye A Napolitano CM Lerner RM. Intentional self-regulation in middle adolescence: the emerging role of loss-based selection in positive youth development. J Youth Adolesc. (2010) 39:764–82. doi: 10.1007/s10964-010-9537-2, PMID: 20424900

[15] Zimmerman SM Phelps E Lerner RM. Positive and negative developmental trajectories in U.S. adolescents: where the positive youth development perspective meets the deficit model. Res Hum Dev. (2008) 5:153–65. doi: 10.1080/15427600802274001

[16] Burkhard BM Robinson KM Murray ED Lerner RM. The positive youth development perspective In: Hupp S Jewell J, editors. The Encyclopedia of Child and Adolescent Development. Hoboken, NJ: Wiley-Blackwell (2019). 1–12.

[17] Landstedt E Almquist YB Eriksson M Hammarström A. Disentangling the directions of associations between structural social capital and mental health: longitudinal analyses of gender, civic engagement and depressive symptoms. Soc Sci Med. (2016) 163:135–43. doi: 10.1016/j.socscimed.2016.07.005, PMID: 27423294

[18] Ballard PJ Hoyt LT Pachucki MC. Impacts of adolescent and young adult civic engagement on health and socioeconomic status in adulthood. Child Dev. (2019) 90:1138–54. doi: 10.1111/cdev.12998, PMID: 29359473

[19] Wray-Lake L Shubert J Lin L Starr LR. Examining associations between civic engagement and depressive symptoms from adolescence to young adulthood in a national U.S. sample. Appl Dev Sci. (2019) 23:119–31. doi: 10.1080/10888691.2017.1326825

[20] Chan RCH Mak WWS. Empowerment for civic engagement and well-being in emerging adulthood: evidence from cross-regional and cross-lagged analyses. Soc Sci Med. (2020) 244:112703. doi: 10.1016/j.socscimed.2019.112703, PMID: 31852582

[21] Reiss F Meyrose AK Otto C Lampert T Klasen F Ravens-Sieberer U. Socioeconomic status, stressful life situations and mental health problems in children and adolescents: results of the German BELLA cohort-study. PLoS One. (2019) 14:e0213700. doi: 10.1371/journal.pone.0213700, PMID: 30865713 PMC6415852

[22] Hammami N Azevedo Da Silva M Elgar FJ. Trends in gender and socioeconomic inequalities in adolescent health over 16 years (2002-2018): Findings from the Canadian Health Behaviour in School-aged Children study. Évolution des inégalités en santé chez les adolescents selon le genre et le statut socioéconomique sur 16 ans (2002 à 2018):résultats de l’Enquête sur les comportements de santé des jeunes d’âge scolaire. Health Promot Chron Dis Prev Can Res Policy Pract. (2022) 42:68–78. doi: 10.24095/hpcdp.42.2.03 PMC8935898 35170931

[23] Campbell OLK Bann D Patalay P. The gender gap in adolescent mental health: a cross-national investigation of 566,829 adolescents across 73 countries. SSM Popul Health. (2021) 13:100742. doi: 10.1016/j.ssmph.2021.100742, PMID: 33748389 PMC7960541

[24] Qvist H-PY Folkestad B Fridberg T Lundåsen SW. Trends in volunteering in Scandinavia In: Henriksen L Strømsnes K Svedberg L, editors. Civic Engagement in Scandinavia. Nonprofit and Civil Society Studies (an International Multidisciplinary Series). Cham: Springer (2019). 67–94.

[25] Schulz W. Ainley J. Fraillon J. Losito B. Agrusti G. Friedman T. (2018). Becoming citizens in a changing world: IEA international civic and citizenship education study 2016 international report. Springer, Cham.

[26] Hegna K. Young citizenship: civic engagement and participation in four Nordic countries In: Strand T, editor. Rethinking Ethical-Political Education. Contemporary Philosophies and Theories in Education, vol. 16. Cham: Springer (2020). 13–28.

[27] Hafstad GS Sætren SS Wentzel-Larsen T Augusti EM. Adolescents' symptoms of anxiety and depression before and during the Covid-19 outbreak—a prospective population-based study of teenagers in Norway. Lancet Reg Health Eur. (2021) 5:100093. doi: 10.1016/j.lanepe.2021.100093, PMID: 34557820 PMC8454857

[28] Ekeberg Ø Hem E. Why is the suicide rate not declining in Norway? Tidsskrift Den Norske Legeforen. (2019) 139. doi: 10.4045/tidsskr.18.0943 31429253

[29] Norwegian Institute of Public Health (2019). “Quality of life and mental health among children and adolescents in Norway” in Public health report—Health status in Norway [online document]. Institute of Public Health, Oslo. Available at: https://www.fhi.no/en/op/hin/mental-health/mental-health-children-adolescents/ (Accessed April 29, 2023)

[30] Howard MC Hoffman ME. Variable-centered, person-centered, and person-specific approaches: where theory meets the method. Organ Res Methods. (2018) 21:846–76. doi: 10.1177/1094428117744021

[31] Wray-Lake L Metzger A Syvertsen AK. Testing multidimensional models of youth civic engagement: model comparisons, measurement invariance, and age differences. Appl Dev Sci. (2017) 21:266–84. doi: 10.1080/10888691.2016.1205495

[32] Larsen T Urke HB Holsen I Anvik CH Olsen T Waldahl RH. COMPLETE–a school-based intervention project to increase completion of upper secondary school in Norway: study protocol for a cluster randomized controlled trial. BMC Public Health. (2018) 18:1–12. 10.1186/s12889-018-5241-z PMC5845360 29523124

[33] Tambs K Moum T. How well can a few questionnaire items indicate anxiety and depression? Acta Psychiatr Scand. (1993) 87:364–7. doi: 10.1111/j.1600-0447.1993.tb03388.x, PMID: 8517178

[34] Gjerde LC Røysamb E Czajkowski N Reichborn-Kjennerud T Ørstavik RE Kendler KS. Strong genetic correlation between interview-assessed internalizing disorders and a brief self-report symptom scale. Twin Res Hum Genet. (2011) 14:64–72. doi: 10.1375/twin.14.1.64, PMID: 21314257 PMC3081885

[35] Skrove M Romundstad P Indredavik MS. Resilience, lifestyle and symptoms of anxiety and depression in adolescence: the young-HUNT study. Soc Psychiatry Psychiatr Epidemiol. (2013) 48:407–16. doi: 10.1007/s00127-012-0561-2, PMID: 22872359

[36] Strand BH Dalgard OS Tambs K Rognerud M. Measuring the mental health status of the Norwegian population: a comparison of the instruments SCL-25, SCL-10, SCL-5 and MHI-5 (SF-36). Nord J Psychiatry. (2003) 57:113–8. doi: 10.1080/08039480310000932 12745773

[37] Clarke A Friede T Putz R Ashdown J Martin S Blake A. Warwick-Edinburgh mental well-being scale (WEMWBS): validated for teenage school students in England and Scotland. A mixed methods assessment. BMC Public Health. (2011) 11:487. doi: 10.1186/1471-2458-11-487, PMID: 21693055 PMC3141456

[38] Ringdal R Bradley Eilertsen M-E Bjørnsen HN Espnes GA Moksnes UK. Validation of two versions of the Warwick-Edinburgh mental well-being scale among Norwegian adolescents. Scand J Public Health. (2018) 46:718–25. doi: 10.1177/1403494817735391, PMID: 29017402

[39] Tennant R Hiller L Fishwick R Platt S Joseph S Weich S. The Warwick-Edinburgh mental well-being scale (WEMWBS): development and UK validation. Health Qual Life Outcomes. (2007) 5:63. doi: 10.1186/1477-7525-5-63, PMID: 18042300 PMC2222612

[40] Hauch D Fjorback LO Juul L. Psychometric properties of the short Warwick-Edinburgh mental well-being scale in a sample of Danish schoolchildren. Scand J Public Health. (2022) 14034948221110002:140349482211100. doi: 10.1177/14034948221110002 35855556

[41] Hunter SC Houghton S Wood L. Positive mental well-being in Australian adolescents: evaluating the Warwick-Edinburgh mental well-being scale. Educ Dev Psychol. (2015) 32:93–104. doi: 10.1017/edp.2015.12

[42] Köhler H. Weber S. Brese F. Schulz W. Carstens R. (2018). ICCS 2016 user guide for the international database. Amsterdam: IEA. Available at: https://www.iea.nl/sites/default/files/2019-05/ICCS2016_IDB_User_Guide.pdf (Accessed April 28, 2023).

[43] Iversen AC Holsenv I. Inequality in health, psychosocial resources and health behavior in early adolescence: the influence of different indicators of socioeconomic position. Child Indic. Res. (2008) 1:291–302.

[44] Elgar FJ McKinnon B Torsheim T Schnohr CW Mazur J Cavallo F. Patterns of socioeconomic inequality in adolescent health differ according to the measure of socioeconomic position. Soc Indic Res. (2016) 127:1169–80. doi: 10.1007/s11205-015-0994-6

[45] Nagin DS. Analyzing developmental trajectories: a semiparametric, group-based approach. Psychol Methods. (1999) 4:139–57. doi: 10.1037/1082-989X.4.2.139 11285809

[46] Nylund KL Asparouhov T Muthén BO. Deciding on the number of classes in latent class analysis and growth mixture modeling: a Monte Carlo simulation study. Struct Equ Model. (2007) 14:535–69. doi: 10.1080/10705510701575396

[47] Lo Y Mendell NR Rubin DB. Testing the number of components in a normal mixture. Biometrika. (2001) 88:767–78. doi: 10.1093/biomet/88.3.767

[48] Muthén LK Muthén BO. Mplus User’s Guide. 8th ed. Los Angeles, CA: Muthén and Muthén (1998–2017).

[49] Lerner RM Lerner JV Bowers E Geldhof GJ. Positive youth development: a relational developmental systems model In: Overton WF Molenaar PC, editors. Handbook of Child Psychology and Developmental Science: Theory and Method, vol. 1. 7th ed. Hoboken, NJ: Wiley (2015). 607–51.

[50] Cho A. Byrne J. Pelter Z. (2020). Digital civic engagement by young people. UNICEF Office of Global Insight and Policy, New York, NY, USA.

[51] Bronfenbrenner U Morris PA. The bioecological model of human development In: Damon W Lerner RM, editors. Handbook of Child Psychology: Vol. 1. Theoretical Models of Human Development. 6th Edn. Hoboken, NJ: John Wiley & Sons Inc (2006). 793–828.

[52] Navarro J. L. Tudge J. R. H. (2022). Technologizing Bronfenbrenner: neo-ecological theory. Curr Psychol. 1–17. doi: 10.1007/s12144-022-02738-3 [Epub ahead of print]. PMC8782219 35095241

[53] Weinberg D Stevens GWJM Duinhof EL Finkenauer C. Adolescent socioeconomic status and mental health inequalities in the Netherlands, 2001-2017. Int J Environ Res Public Health. (2019) 16:3605. doi: 10.3390/ijerph16193605, PMID: 31561487 PMC6801857

[54] Lowthian E Page N Melendez-Torres GJ Murphy S Hewitt G Moore G. Using latent class analysis to explore complex associations between socioeconomic status and adolescent health and well-being. J Adolesc Health. (2021) 69:774–81. doi: 10.1016/j.jadohealth.2021.06.013, PMID: 34275658 PMC9225957

[55] Daly. Cross-national and longitudinal evidence for a rapid decline in life satisfaction in adolescence. J Adolesc. (2022) 94:422–34. doi: 10.1002/jad.12037, PMID: 35390206

